# Supplementary material for: Environmental attitude and affective-motivational beliefs towards sustainability of secondary school children in Germany and their associations with gender, age, school type, socio-economic status and time spent in nature
Source: PLoS One. 2024 May 1;19(5):e0296327. doi: 10.1371/journal.pone.0296327 (PMC11062540; doi:10.1371/journal.pone.0296327)
Supplement: S3 Fig — UTL, utilization; PRE, preservation; AMBTS, affective-motivational beliefs towards sustainability. (PDF) [file pone.0296327.s003.pdf]

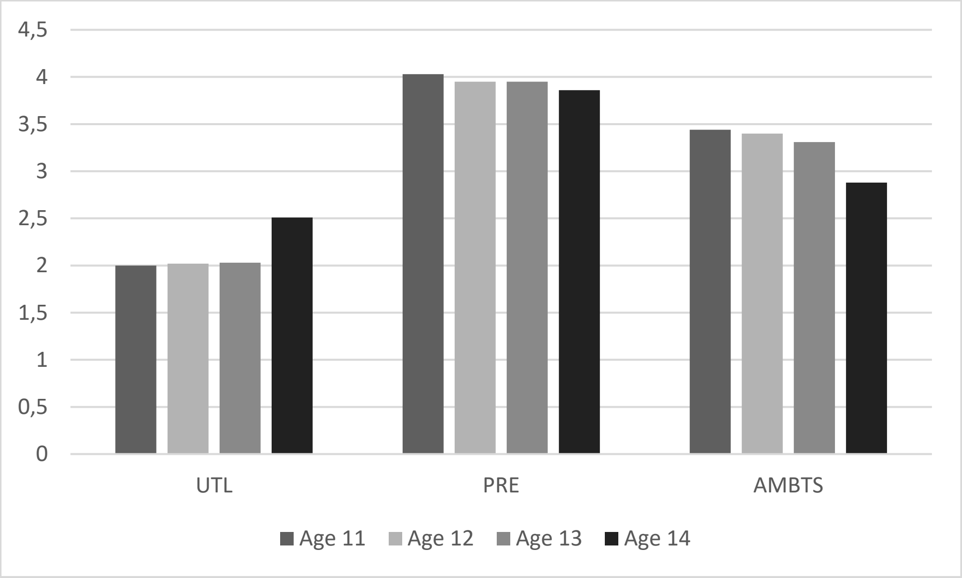

S3 Fig. Graphical representaion of mean values according to age.

UTL, utilization; PRE, preservation; AMBTS, affective-motivational beliefs towards sustainability.
