# Supplementary material for: Environmental attitude and affective-motivational beliefs towards sustainability of secondary school children in Germany and their associations with gender, age, school type, socio-economic status and time spent in nature
Source: PLoS One. 2024 May 1;19(5):e0296327. doi: 10.1371/journal.pone.0296327 (PMC11062540; doi:10.1371/journal.pone.0296327)
Supplement: S4 Fig — UTL, utilization; PRE, preservation; AMBTS, affective-motivational beliefs towards sustainability; TSIN, time spent in nature. The number after TSIN represents the item response categories regarding the amount of time per week spent in nature: 1, half an hour or less; 2, between half an hour and two hours; 3, between two and three and a half hours; 4, three and a half hours or more. (PDF) [file pone.0296327.s004.pdf]

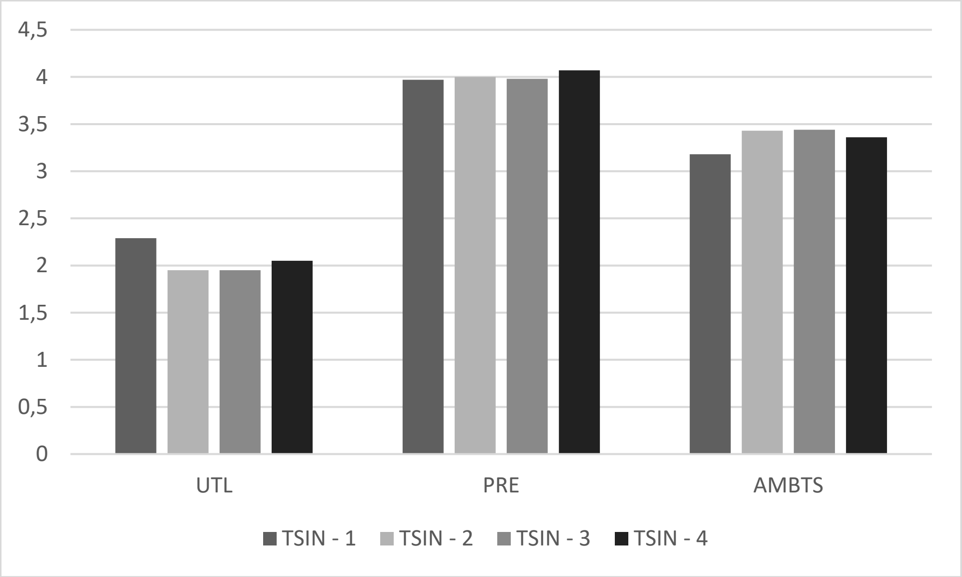

S4 Fig. Graphical representaion of mean values according to time spent in nature.

UTL, utilization; PRE, preservation; AMBTS, affective-motivational beliefs towards sustainability; TSIN, time spent in nature. The number after TSIN represents the item response categories regarding the amount of time per week spent in nature: 1, half an hour or less; 2, between half an hour and two hours; 3, between two and three and a half hours; 4, three and a half hours or more.
