# Supplementary material for: Environmental attitude and affective-motivational beliefs towards sustainability of secondary school children in Germany and their associations with gender, age, school type, socio-economic status and time spent in nature
Source: PLoS One. 2024 May 1;19(5):e0296327. doi: 10.1371/journal.pone.0296327 (PMC11062540; doi:10.1371/journal.pone.0296327)
Supplement: S1 File — (PDF) [file pone.0296327.s005.pdf]

Professur für Sport- und Gesundheitsdidaktik
Fakultät für Sport- und Gesundheitswissenschaften
Technische Universität München

**Die folgenden Fragen betreffen Deine Einstellung zur Umwelt.**					
	Völlig richtig	Ziemli ch richtig	unents chiede n	Ziemli ch falsch	Völlig falsch
Wir müssen mehr Straßen bauen, damit die Menschen aufs Land fahren können.	☐	☐	☐	☐	☐
Die Natur ist immer in der Lage, sich aus eigener Kraft wiederherzustellen.	☐	☐	☐	☐	☐
Unser Planet hat unbegrenzte Ressourcen.	☐	☐	☐	☐	☐
Wir müssen keine Gebiete schützen, um bedrohte Arten zu bewahren.	☐	☐	☐	☐	☐
Die Menschen machen sich zu viele Gedanken über Umweltverschmutzung.	☐	☐	☐	☐	☐
Die stille Natur draußen macht mich ängstlich.	☐	☐	☐	☐	☐
Wir müssen Wälder roden, um Nutzpflanzen (z.B. Getreide) anzubauen.	☐	☐	☐	☐	☐
Menschen haben nicht das Recht, die Natur zu ändern, wie sie es für richtig halten.	☐	☐	☐	☐	☐
Menschen sind nicht wichtiger als andere Lebewesen.	☐	☐	☐	☐	☐
Um Wasser zu sparen, dusche ich anstatt zu baden.	☐	☐	☐	☐	☐
Schmutziger Industrierauch aus Kaminen macht mich wütend.	☐	☐	☐	☐	☐
Die Menschheit wird aussterben, wenn wir nicht im Einklang mit der Natur leben.	☐	☐	☐	☐	☐
Nicht nur Pflanzen und Tiere von wirtschaftlicher Bedeutung sollten geschützt werden.	☐	☐	☐	☐	☐

The following questions concern your attitude towards the environment.					
	Totally right	Fairly right	Undecided	Fairly wrong	Totally wrong
We need to build more roads so people can go to the countryside.	☐	☐	☐	☐	☐
Nature is always able to restore itself on its own.	☐	☐	☐	☐	☐
Our planet has unlimited resources.	☐	☐	☐	☐	☐
We don't have to protect areas to conserve endangered species.	☐	☐	☐	☐	☐
People worry too much about pollution.	☐	☐	☐	☐	☐
The silent nature outside makes me anxious.	☐	☐	☐	☐	☐
We have to clear forests to grow crops (e.g. grain).	☐	☐	☐	☐	☐
Humans do not have the right to change nature as they see fit.	☐	☐	☐	☐	☐
People are no more important than other living beings.	☐	☐	☐	☐	☐
To save water, I shower instead of bathing.	☐	☐	☐	☐	☐
Dirty industrial smoke from chimneys infuriates me.	☐	☐	☐	☐	☐
Humanity will die out if we don't live in harmony with nature.	☐	☐	☐	☐	☐
Not only plants and animals of economic importance should be protected.	☐	☐	☐	☐	☐
