# Supplementary material for: Environmental attitude and affective-motivational beliefs towards sustainability of secondary school children in Germany and their associations with gender, age, school type, socio-economic status and time spent in nature
Source: PLoS One. 2024 May 1;19(5):e0296327. doi: 10.1371/journal.pone.0296327 (PMC11062540; doi:10.1371/journal.pone.0296327)
Supplement: S2 File — (PDF) [file pone.0296327.s006.pdf]

Professur für Sport- und Gesundheitsdidaktik
Fakultät für Sport- und Gesundheitswissenschaften
Technische Universität München

Die nächsten Fragen befassen sich mit deiner Einstellung zu nachhaltiger Entwicklung.				
	Stimme nicht zu	Stimme eher nicht zu	Stimme eher zu	Stimme zu
Ich interessiere mich für die Ursachen des Klimawandels.	☐	☐	☐	☐
Mir ist es wichtig, dass ich nur Dinge kaufe, die unter guten Bedingungen für die Umwelt und die Arbeiter*innen hergestellt wurden.	☐	☐	☐	☐
Das Aussterben vieler Tier- und Pflanzenarten macht mich traurig.	☐	☐	☐	☐
Wenn ich von Autos höre, die viel Sprit verbrauchen und viel Abgase ausstoßen, ärgere ich mich.	☐	☐	☐	☐
Informationen über Zusammenhänge in der Natur, das Leben in anderen Ländern und wie Dinge in der Welt zusammenhängen langweilen mich.	☐	☐	☐	☐
Ich finde es wichtig, dass Deutschland als reiches Land den ärmeren Ländern hilft.	☐	☐	☐	☐
Ich finde, dass unser Verhalten so sein sollte, dass auch die Menschen, die nach mir geboren werden, gut leben können.	☐	☐	☐	☐
Es sollte alle Menschen interessieren, wie wir und die Menschen in anderen Ländern gut zusammenleben können.	☐	☐	☐	☐
Ich finde es wichtig, dass sich Jung und Alt und Menschen aus anderen Ländern für die Probleme der jeweils anderen interessieren.	☐	☐	☐	☐
Ich finde es wichtig, mich für eine gerechte Gesellschaft einzusetzen.	☐	☐	☐	☐
Ich finde es wichtig, mich für den Schutz von Tieren und Pflanzen einzusetzen.	☐	☐	☐	☐
Ich finde es wichtig, dass die Dinge, die ich esse, aus fairem Handel kommen.	☐	☐	☐	☐
Ich finde es wichtig, dass es bei uns faire und gerechte Arbeitsbedingungen gibt.	☐	☐	☐	☐
Es macht mich traurig, dass so viele Lebensräume zerstört werden.	☐	☐	☐	☐
Ich finde es wichtig, dass es in anderen Ländern keine Kinderarbeit gibt und die Menschen für ihre Arbeit gerecht bezahlt werden.	☐	☐	☐	☐
Ich finde es wichtig, mehr über Menschen aus anderen Ländern und ihre Probleme zu erfahren.	☐	☐	☐	☐

The next questions deal with your attitude towards sustainable development.				
	I do not agree	I rather disagree	I rather agree	I do agree
I am interested in the causes of climate change.	☐	☐	☐	☐
It is important to me that I only buy things that have been manufactured under good conditions for the environment and the workers.	☐	☐	☐	☐
The extinction of many animal and plant species makes me sad.	☐	☐	☐	☐
When I hear about cars that use a lot of fuel and emit a lot of exhaust fumes, I get annoyed.	☐	☐	☐	☐
Information about connections in nature, life in other countries and how things in the world are connected bores me.	☐	☐	☐	☐
I think it's important that Germany, as a rich country, helps poorer countries.	☐	☐	☐	☐
I think that our behavior should be such that the people who will be born after me can also live well.	☐	☐	☐	☐
Everyone should be interested in how we and people in other countries can live well together.	☐	☐	☐	☐
I think it's important that young and old and people from other countries are interested in each other's problems.	☐	☐	☐	☐
I think it's important to work for a just society.	☐	☐	☐	☐
I think it's important to work to protect animals and plants.	☐	☐	☐	☐
I think it's important that the things I eat are fair trade.	☐	☐	☐	☐
I think it is important that we have fair and just working conditions.	☐	☐	☐	☐
It saddens me that so many habitats are being destroyed.	☐	☐	☐	☐
I think it's important that there is no child labor in other countries and that people are paid fairly for their work.	☐	☐	☐	☐
I think it's important to learn more about people from other countries and their problems.	☐	☐	☐	☐
