# Supplementary material for: Environmental attitude and affective-motivational beliefs towards sustainability of secondary school children in Germany and their associations with gender, age, school type, socio-economic status and time spent in nature
Source: PLoS One. 2024 May 1;19(5):e0296327. doi: 10.1371/journal.pone.0296327 (PMC11062540; doi:10.1371/journal.pone.0296327)
Supplement: Attachment — Submitted filename: Response to Reviewers.docx [file pone.0296327.s008.docx]

*Dear Editor, dear Reviewers,*

*We would like to thank you for your helpful comments. We believe that the quality of our manuscript has improved by implementing the suggestions and comments you made. Please find our response to the comments below.* *When we refer to lines, we always refer to the file labeled "Revised Manuscript with Track Changes". All our responses are written in italics. We hope we have addressed all your concerns and comments to your satisfaction.*

Journal requirements:

When submitting your revision, we need you to address these additional requirements.

1. Please ensure that your manuscript meets PLOS ONE's style requirements, including those for file naming. The PLOS ONE style templates can be found at

https://journals.plos.org/plosone/s/file?id=wjVg/PLOSOne_formatting_sample_main_body.pdf and

https://journals.plos.org/plosone/s/file?id=ba62/PLOSOne_formatting_sample_title_authors_affiliations.pdf

2. You indicated that you had ethical approval for your study. In your Methods section, please ensure you have also stated whether you obtained consent from parents or guardians of the minors included in the study or whether the research ethics committee or IRB specifically waived the need for their consent.

*Thank you for this note. We obtained consent from parents or legal guardians and included a statement in the Methods section. Please see lines 177 - 178.*

3. PLOS requires an ORCID iD for the corresponding author in Editorial Manager on papers submitted after December 6th, 2016. Please ensure that you have an ORCID iD and that it is validated in Editorial Manager. To do this, go to ‘Update my Information’ (in the upper left-hand corner of the main menu), and click on the Fetch/Validate link next to the ORCID field. This will take you to the ORCID site and allow you to create a new iD or authenticate a pre-existing iD in Editorial Manager. Please see the following video for instructions on linking an ORCID iD to your Editorial Manager account: <https://www.youtube.com/watch?v=_xcclfuvtxQ>

*Thank you for the instructions. I added my ORCID iD to my Editorial Manager account.*

4. Your ethics statement should only appear in the Methods section of your manuscript. If your ethics statement is written in any section besides the Methods, please move it to the Methods section and delete it from any other section. Please ensure that your ethics statement is included in your manuscript, as the ethics statement entered into the online submission form will not be published alongside your manuscript.

*Thank you for the comment. The ethics statement now only appears in the Methods section.*

Additional Editor Comments:

We apologise for delays in getting reviewer feedback to you. We have now obtained the required number of reviews for your manuscript and invite you to revise addressing concerns from the reviewers.

Reviewers' comments:

Reviewer's Responses to Questions

**Comments to the Author**

1. Is the manuscript technically sound, and do the data support the conclusions?

The manuscript must describe a technically sound piece of scientific research with data that supports the conclusions. Experiments must have been conducted rigorously, with appropriate controls, replication, and sample sizes. The conclusions must be drawn appropriately based on the data presented.

Reviewer #1: Yes

Reviewer #2: Yes

2. Has the statistical analysis been performed appropriately and rigorously?

Reviewer #1: No

Reviewer #2: Yes

*We substantially revised the statistical analysis according to the reviewers’ comments. We think that these changes benefited the quality of the findings and the discussion of the findings. Thank you again for your helpful suggestions.*

3. Have the authors made all data underlying the findings in their manuscript fully available?

The PLOS Data policy requires authors to make all data underlying the findings described in their manuscript fully available without restriction, with rare exception (please refer to the Data Availability Statement in the manuscript PDF file). The data should be provided as part of the manuscript or its supporting information, or deposited to a public repository. For example, in addition to summary statistics, the data points behind means, medians and variance measures should be available. If there are restrictions on publicly sharing data—e.g. participant privacy or use of data from a third party—those must be specified.

Reviewer #1: No

Reviewer #2: Yes

*Thank you for your feedback. The data set (supplementary file) is included. Additionally, we now included the translated questionnaires.*

4. Is the manuscript presented in an intelligible fashion and written in standard English?

PLOS ONE does not copyedit accepted manuscripts, so the language in submitted articles must be clear, correct, and unambiguous. Any typographical or grammatical errors should be corrected at revision, so please note any specific errors here.

Reviewer #1: Yes

Reviewer #2: Yes

5. Review Comments to the Author

Reviewer #1: Review of the article „Environmental attitude and affective-motivational beliefs towards sustainability of secondary school children in Germany and their associations with gender, age, school type, socioeconomic status and time spent in nature”

The article addresses an interesting and relevant topic. It describes sustainability and environmental attitudes in children and adolescents in Germany and investigates associations with sociodemographic characteristics and time spent in nature. Socioeconomic status and education are suggested to mainly shape these attitudes in children and adolescents.

The article is well written. However, theoretical aspects might be shortened, while descriptions of the outcomes in the sample should be more detailed. Furthermore, the analyses could be homogenized.

General comment:
You write at several point in the manuscript that your findings suggest a tentative recommendation to spend at least half an hour per week in nature. I do not believe that this would improve sustainability and environmental attitude. As you write in the Discussion, it is more probable that sustainability/environmental attitudes and spending time outdoors are both affected by a third factor, e.g., socioeconomic status of the family. Therefore, I would completely remove recommendations regarding time spending in nature.

*Thank you for your general comments. Based on the revision of our manuscript, we would like to address some of the general aspects that you mentioned here. In response to your suggestion, we homogenized the statistical analysis and now only perform linear regression analyses. As you mentioned in your comment, the variable ‘time spent in nature’ cannot really be considered metric. Therefore, it was dummy-coded in order to include it in the regression analysis. The multiple regression analysis indicated that time spent in nature does play a statistically significant role, also when socio-economic status and school type are simultaneously included in the analysis as further predictors. We would like to thank you again for your important recommendation regarding the statistical analysis since it has led to more accurate findings. Please see the results and discussion sections and our responses to your specific comments below for further information.*

Please avoid statements on causality (e.g., abstract, line 25, “predictor”).

*Thank you for this suggestion. Given our study design, we try to avoid to suggest any causal relationships and rather refer to terms like ‘associated to’ etc. wherever possible. The term ’predictor’ is only used within the framework of regression analyses to differentiate predictor and criterion variables.*

For someone being no expert in the assessment of sustainability and environmental attitude, it is difficult to understand was actually assessed and how the students behaved. Could you provide a copy of the questionnaire items in the supplement? And maybe a figure on findings in your sample (not only means)?

*Thank you for the helpful comment. We added our (translated) questionnaire as a supplementary file. The results section was largely revised (see also our responses to your other comments) and now includes tables where the results are visible at a glance. However, we were not sure what type of figures you had in mind. We have now added four figures as supporting file that illustrate the level of environmental attitude and affective-motivational beliefs towards sustainability in subgroups according to the different explanatory variables. If this is not what you had in mind when writing the comment, please let us know and we will add the figures accordingly.*

In some sentences, the English does not seem to be correct. Can a native speaker check the manuscript?

*Thank you very much for this suggestion. A native speaker colleague checked the language again.*

Abstract:
I do not understand the last sentence (lines 34ff). I would remove it.

*Thank you for this comment. We checked the sentence and removed it.*

Introduction:
Line 42f: I suggest removing “[…] and created well understandable graphics”. This is not really relevant, is it?

*Thank you for this comment. We totally agree and deleted this part of the sentence.*

Line 82: What is meant by “Knowledge about a certain object alone”? Which object?

*Thank you very much for your comment. We changed “certain object” into “environmental topics”.*

Regarding environmental attitude, you mention different categorizations, e.g., “functions and structures“ (line 88) and “utilization and preservation” (line 96f). This is misleading.

*Thank you for your comment. We have rewritten the first sentence in this paragraph so that the misunderstanding does not occur in the text.* *Please see lines 99 - 100.*

Line 104ff: I suggest starting this paragraph with the term “affective-motivational beliefs” as part of sustainability attitudes (not with the term “sustainability attitudes”) as this is the variable you are assessing. Otherwise, it is misleading.

*Thank you for your comment. Unfortunately, we cannot implement your suggestion, since the beginning of the paragraph is about Attitude in general, and then follows the classification of affective-motivational beliefs towards sustainability. These are the closest to sustainability attitudes in the scientific context.*

The introduction is too long. I suggest shortening explanations on the constructs. Rather give concrete examples.

*Thank you very much for this comment. After discussing the idea of replacing the explanations of the constructs with concrete examples, we decided to not change the introduction. Understanding the constructs, what they refer to and what they do not refer to, is a rather complex task. Therefore, we find it very important to give elaborate explanations on the constructs to make sure that they are correctly understood. We hope that you are fine with this approach.*

Materials and methods:
Instead of using the term Gymnasium and Realschule (which are only known in Germany) please use a more general term, e.g., “higher” versus “lower” secondary school education. Also, you don’ have to mention that there exist four types of secondary school (line 170). As you did only distinguish two, information on 2 others in misleading.

*Thank you for these comments. We changed the terms in the manuscript into higher and lower secondary schools (HSS/LSS) and deleted the sentence about the four school types in Germany.*

Please provide percentages for children from lower and higher secondary schools.

*Thank you for this comment. We added the percentages. See line 174.*

Why are there so much more girl than boys in your sample?

*In Bavaria, there are no coeducational physical education classes. Female teachers teach girls and male teachers teach boys. The female teachers were more motivated to take part in the study than the male teachers were. Accordingly, more data was collected from girls.*

Line 192f: Please provide values/ranges for high loading on the latent factor and for the low cross-loading.

*Thank you for this comment. We have reworded the sentence so that it can now be read out which loadings are present on the latent factor and for the cross-loading*. *Please see lines 210 - 212.*

Why don’t you perform linear regressions only? Why do you apply correlations, ANOVAs, and regressions? And sometimes ANOVAs AND linear regression? Using (only) regressions also for univariate associations would make results more comparable. Also, regression coefficients from uni- and multivariate analyses could be compared more easily.

*Thank you for this comment. We have completely revised the results section and made new calculations. You will now find univariate regression analyses in the results section in addition to the multiple regression analysis. We also calculated regressions for "time spent in nature" by creating dummy variables. Thus, the results are now more comparable.*

Did you report standardized or non-standardized regression coefficients?

*In the revised results section, we now report both standardized and non-standardized regression coefficients.*

Results:
Table 1: Please add the possible range. This makes it easier to interpret the mean values. What does the last line of the Table tells us? Is it necessary? Shouldn’t it be the same as in Table 2 (which is not the case)? Please explain UTL and PRE in the caption. What do the * mean? What was compared (using which analyses)?

*Thank you for the comments and questions. The range is described in the methods section (please see lines 202 – 205 and 218 - 219). We revised Table 1, so that you can see mean values and standard deviations. The last line of the table and the * were removed (not useful anymore due to the substantial revision of the methods section) and a note is added, where every abbreviation is explained.*

Please remove subsection headings 3.2.1-3.2.4.

*Thank you for this comment. We removed the subsections.*

Line 281f: time spend in nature is not really metric. However, it seems as if you included it as a metric independent variable into the regression. I think, that’s not the best way to analyze these data.

*Thank you for this comment. As mentioned earlier, we now use dummy variables for time spent in nature to be able to include the variable in linear regression analyses.*

Discussion
Line 336f: Regarding age, you performed a correlation, didn’t you? Here, it seems as if you compared age groups. This is misleading.

*Thank you for your comment. We now refer to Table 1 in the text and have added a paragraph so that this misunderstanding no longer prevails. Please see lines 417 – 428.*

Lines 419ff: The paragraph on internal consistency and factor loading is too long. These details are not needed in the limitations section.

*Thank you for this comment. We agree that the statements on internal consistency and factor analysis is quite long. However, we consider these aspects to be of high importance when conducting a questionnaire assessment. Therefore, we wanted to discuss the limitation of low internal consistency in more detail and, most importantly, transparently.*

Figure
Is this figure really necessary? A Figure on the outcomes would be more appropriate.

*Thank you for your comment. As we wrote in our response to one of your comments above, we added four figures and removed the one referring to ISEI.*

Reviewer #2: My first reaction with the paper was positive, when reading the harvest message, the sample sizes and the application of established scales. Many usual mistakes of studies in our field seemed omitted. However when reading a second, a third time, inconsistencies appeared. In consequence, the manuscript seems in a pre-mature stage and needs substantial improvement before accepting to publication in Plos1.

Let me go more into detail by limiting my queries to 10 issues:
1) The sample’s age distribution is quite unbalanced (and needs discussion).

*Thank you for this comment. We are aware of the unequal distribution and for this reason, we have written a section in the Discussion that refers precisely to this. Please see lines 417 – 428.*

2) Table 2: The item itself is quite simple and maybe not valid, as time ranges for that age group might be overrated. Additionally, a age correction as covariate should be taken into consideration.

*Thank you very much for this hint. We are aware of the possible inaccuracy of time estimates of adolescents and have therefore designed the categories rather broadly, so that at least the assignment to these broader categories is relatively reliable. In our opinion, a more reliable and at the same time economical and reasonable way of assessing the construct was not possible. Diaries would certainly be more reliable, but also more time-consuming, and thus less economical. Due to probably emerging compliance problems, the sample size with regard to this variable would then have been substantially reduced.*

*Thank you for your suggestion regarding a potential age correction. Since we have conducted a multiple regression analysis, an age correction is automatically done since all predictor variables are included simultaneously.*

3) Knowledge, the manuscript is citing #31, but not differentiating the types of knowledge. The authors seemingly just concentrate on factual knowledge or system knowledge. A related application study of that specific model was published 2020 by Maurer & colleagues.

*Thank you for your comment. We have now included the differentiation of the different types of knowledge (please see lines 89 - 92). We subsequently define these in one sentence, as our article is exclusively about environmental attitude and we would like to mention knowledge and behavior only briefly in order to avoid any misunderstandings.*

4) As the authors use 4- and 5-digit response patterns, this needs discussion.

*Thank you for this comment. We added the information about it to the first paragraph in the Discussion section. “We used a 4- and a 5-point Likert scale for affective-motivational beliefs towards sustainability respectively environmental attitude. These unequal scales result from the fact that the original scaled and validated response items were retained.”*

5) The 3.4. sub-chapter needs more elaboration.

*Thank you for your comment. We added a table and revised the text in this paragraph. A more detailed discussion of these results can be found in the Discussion section; please see for example lines 422 - 426.*

6) Conclusion: the first 2 sentences are just redundant

*Thank you for this comment. The first sentence summarizes the study that all values are higher compared to other studies. At the same time, however, a difference can be seen within our data: The scores of children with low socioeconomic status and lower formal level are lower than those of the other groups. Thus, there is a more intensive need for environmental education for these groups. We have tried to resolve this with formulations that are more precise. See lines 527 - 530.*

7) Paper 34 is insufficiently cited, therefore approaching that paper is not possible. In my vague memory from conferences, the cited scale of that group was not convincing.

*Thank you for this hint. We corrected the citation.*

*In the article (Waltner et al., 2019), Cronbach’s alpha for the scale we used was .84 and the conclusion of the paper is “According to the presented results, the questionnaire can be approved for practical application to measure different dimensions of sustainability competencies”. When we started to plan and conduct our data assessment in 2019, there were no validated scales available to assess sustainability attitude or similar constructs. There were also no articles that criticized the scale or even recommended not to use it. This has not changed until today, and this despite the fact that, in the meantime, the validation study has been frequently cited.*

8) Reference #60 seems to present an internal report rather than a peer-reviewed paper.

*Thank you for this comment. It is a political report summarizing the results of the OECD Pisa study 2018. We added the official PISA 2018 Results for Germany as reference. The authors did not find a peer-reviewed paper in the context of concentration and learning experience in German schools.*

9) References are inconsistent anyway: Sometime with written first name, sometimes just with a letter (e.g. #60, 61). Some references seemingly were added to enrich the reference list as they are not integrated in argumentation lines.

*Thank you for this comment. The reference list has been completely revised so that now only last name and the first letter of the first name appear. However, no references had been added to the reference list that would not have appeared in the main text. All references had been inserted in the mansucript via EndNote.*

10) The rooting in the literature body of the last two decades seems quite fragmentary: Especially two studies would have laid more foundation: Bogner & Wiseman (1997) [rural-urban samples] and Baierl et al (2022) [age gradient]. Especially for the latter a trend seems visible (which with higher samples sizes might have reached significance).

*Thank you very much for your comment and literature suggestions. Bogner and Wiseman (1997) is well known by the authors but we could not find a way to include the findings of this article. We did not conduct a rural-urban comparison and the study discusses verbal commitment and environmental behavior, which we did not address in our study. Baierl et al. (2022) fit into the discussion nicely.*

Nevertheless there is hope, my recommendation: Major revisions
