# Supplementary material for: Environmental attitude and affective-motivational beliefs towards sustainability of secondary school children in Germany and their associations with gender, age, school type, socio-economic status and time spent in nature
Source: PLoS One. 2024 May 1;19(5):e0296327. doi: 10.1371/journal.pone.0296327 (PMC11062540; doi:10.1371/journal.pone.0296327)
Supplement: Attachment — Submitted filename: Response to Reviewers_Nov.docx [file pone.0296327.s009.docx]

*Dear Editor, dear Reviewers,*

*We would like to thank you again for your helpful comments. We believe that the quality of our manuscript has improved by implementing the suggestions and comments you made. Please find our response to the comments below.* *When we indicate line numbers, we always refer to the file labeled "Revised Manuscript with Track Changes". All our responses are written in italics. We hope we have addressed all your concerns and comments to your satisfaction.*

**Reviewer 1**

Reviewer #1: Thank you for addressing the comments I made. I have only a few minor comments:

1. Second line within the "Study design and sample" section: You now refer to high and lower secondary school, which is totally correct. Therefore, please remove "Realschule" and "Gymnasium" also at the beginning of this section. "Secondary school" is sufficient at that point.

*Thank you very much for this comment. We removed “Realschule and Gymnasium” in line 177.*

2. I suggest combining Table 2 and Table 3 to one Table (this allows direct comparisons between univariate and multiple regression). For example, put information on Table 2 on the left, and information of Table 3 on the right.

*Thank you very much for this idea. We have combined Table 2 and Table 3 into one Table (now Table 2). The text that refers to the results presented in the table was slightly adapted accordingly.*

3. In the tables, please make clear to which gender/school type the numbers refer to (I think, it should be "male" instead of gender, and higher secondary school instead of school type).

*Thank you for your helpful comment. We translated your hint into another idea. Therefore, we used the notes under Table 2 to indicate how gender and school type were coded so that the readers can interpret the values more easily. This way we could name all predictors according to the general construct and did not have to mix construct names with indications of specific types (male, HSS) of the constructs.*

4. You mention in the Discussion that TSIN was only significantly associated with PRE in multiple regression analysis. However, according to Table 3, the associations with UTL and affective-motivational beliefs towards sustainability were stronger (and significant). Please clarify.

*Thank you very much for this comment. You are completely right. Thank you for bringing this error to our attention. We have corrected the respective part of the discussion and adapted it so that it matches the results, please see lines 403-425 and lines 430-434. Furthermore, we revised the abstract accordingly, please see lines 29-35.*

**Reviewer 2**

*Dear Reviewer,*

*When reading your comments, it became clear that you have apparently and unfortunately read and referred to the line specifications and content of the February 2023 version of our manuscript (the first version that was submitted). In reaction to your comments regarding the originally submitted version, we had addressed your concerns and comments in the penultimate version of our manuscript, which we submitted in August 2023. In the following, we now would like to respond to those comments again in which you (quite logically) expressed your dissatisfaction with the last revision of the manuscript.*

Reviewer #2: 1) The sample’s age distribution is quite unbalanced (and needs discussion)

OK

2) Table 2: The item itself is quite simple and maybe not valid, as time ranges for that age group might be overrated. Additionally, a age correction as covariate should be taken into consideration.

OK

3) Knowledge, the manuscript is citing #31, but not differentiating the types of knowledge. The authors seemingly just concentrate on factual knowledge or system knowledge. A related application study of that specific model was published 2020 by Michaela Maurer and colleagues.

Still pending, but I understand that this is not repairable anymore. Thus OK if you acknowledge this as a limitation

*Thank you for your comment. We had included the differentiation of the different types of knowledge (please see lines 91-94). However, we had subsequently defined these in only one sentence, as our article is exclusively about environmental attitude and we would like to mention knowledge and behavior only briefly in order to avoid any misunderstandings or lead the reader away from the core aspects of the article.*

minor: line 91 (Roczen and colleagues); line 92 (Kaiser and colleagues)

*Thank you for this comment. We have added “colleagues”. Please see lines 104 and 105.*

4) As the authors use 4- and 5-digit response patterns, this needs discussion.

OK

5) The 3.4. sub-chapter needs more elaboration

OK

6) Conclusion: the first 2 sentences are just redundant

Unclear response as in my pdf-version lines 527 – 530 points to reference 40 and 41

*Thank you for this comment. The first sentence of the conclusion refers to the fact that the values of our sample in the constructs of environmental attitude and affective-motivational beliefs towards sustainability tend to be more positive compared to other studies. The second sentence refers to the fact that, at the same time, subgroup-related differences can be seen within our data: The scores of children with low socioeconomic status and lower formal level of education are less positive (in a pro-environmental sense) than those of the other groups. Thus, there is a more intensive need for environmental education for these subgroups. In the penultimate version of the manuscript, we had tried to resolve this problem with formulations that are more precise. Please see lines 473-477 in the most current version.*

7) Paper 34 is insufficiently cited, therefore approaching that paper is not possible. In my vague memory form conferences, the cited scale of that group was not convincing.

Still insufficient, better drop this citation

*Thank you for this comment. We have revised the reference. However, we cannot drop the citation because it is the reference for the PRE and UTL scales that we have used.*

8) Reference #60 seems to present an internal report rather than a peer-reviewed paper.

still insufficiently cited, just drop this citation as this is just an internal report

*Thank you for this comment. We now have removed #62 “*PISA 2018 Grundbildung im internationalen Vergleich – Zusammenfassung“  *as we think that you refer to this reference (reference #62 in the current version was reference #60 in the original version that you have commented on).*

9) References are inconsistent anyway: Sometime with written first name, sometimes just with a letter (e.g. #60, 61).

Some references seemingly were added to enrich the reference list as they are not integrated in argumentation lines

For the new #60, still incorrect, nevertheless I recommend dropping anyway (see 9)

*Thank you for this comment. We dropped it.*

10) The rooting in the literature body of the last two decades seems quite fragmentary: Especially two studies would have laid more foundation: Bogner & Wiseman (1997) [rural-urban samples] and Baierl et al (2022) [age gradient]. Especially for the latter a trend seems visible (which with higher samples sizes might have reached significance).

Agree with the first one, but couldn't locate “Baierl and colleagues” in pointing to age coefficients in the new version

*Thank you for this comment. We had referred to Baierl and colleagues in the penultimate version of the manuscript. Please see lines 360-362in the current version.*

11) New: Figure 2: A lot of space of presenting no difference. How about reducing to two subsample; 30 min or less vs 30 min or more (i.e. difference vs no difference)

*Thank you for this comment. In an earlier version of the manuscript, we had conducted the analysis roughly the way you suggest it to be done. However, in reaction to an advice of one reviewer, we have changed this and used dummy variables for time spent in nature in order to be able to include it in the regression analyses. We really appreciate the benefits that come with this type of analysis as it allows to examine whether variable levels 2 through 4 all differ from variable level 1 and whether there are differences between levels 2, 3 and 4 (when looking at the B values).*

*Apart from that, we have now combined the former version of Table 2 and Table 3 into one table (now Table 2) in order to save space and make comparisons of the results of the univariate and multiple regression analyses easier. This was a suggestion from Reviewer 1. We hope you also agree with this.*
